# Supplementary material for: Evolution of DNA packaging in gene transfer agents
Source: Virus Evol. 2021 Feb 19;7(1):veab015. doi: 10.1093/ve/veab015 (PMC7947584; doi:10.1093/ve/veab015)
Supplement: veab015_Supplementary_Data [file veab015_supplementary_data.zip › Supplementary_Figures_S1_S2_Esterman_et_al.pdf]

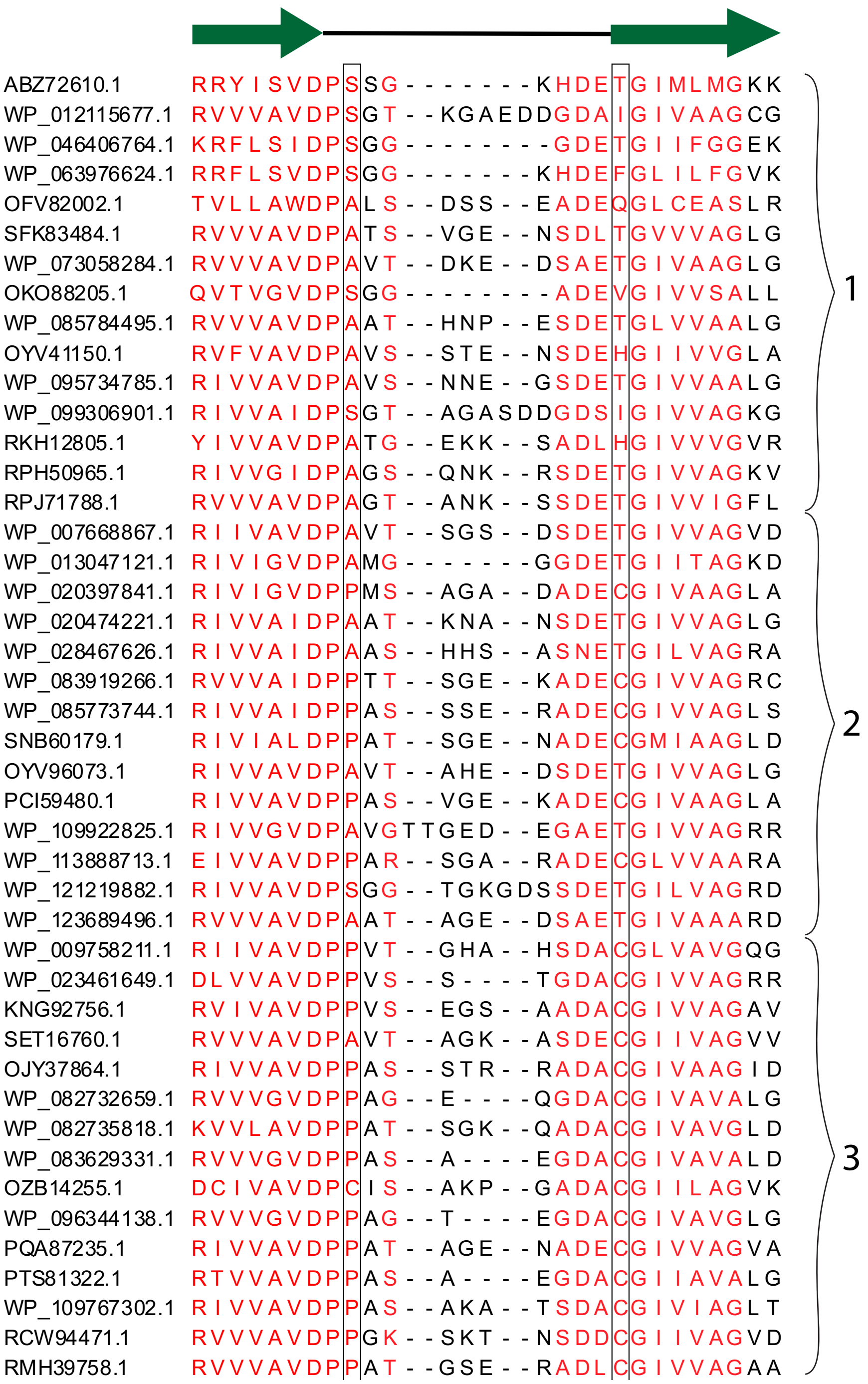

Supplementary Figure S1. Multiple sequence alignment of the TerL region with two conserved sites that differentiate GTA and viral TerLs. The shown selection of representative sequences is designated by their RefSeq or GenBank identifiers. The selected sequences representing the three groups from **Figure 2** (marked by curly braces). The secondary structure of the region is shown above the alignment, where green arrows designate beta strands and the black line indicates a random coil. Alignment sites colored in red contain at least 2 bits of information. The two conserved differentiating sites are outlined by rectangles. The amino acids found in these sites in the full dataset are provided in **Supplementary Table S4**. The depicted region of the protein is highlighted on the TerL protein structure shown in **Supplementary Figure S2**).

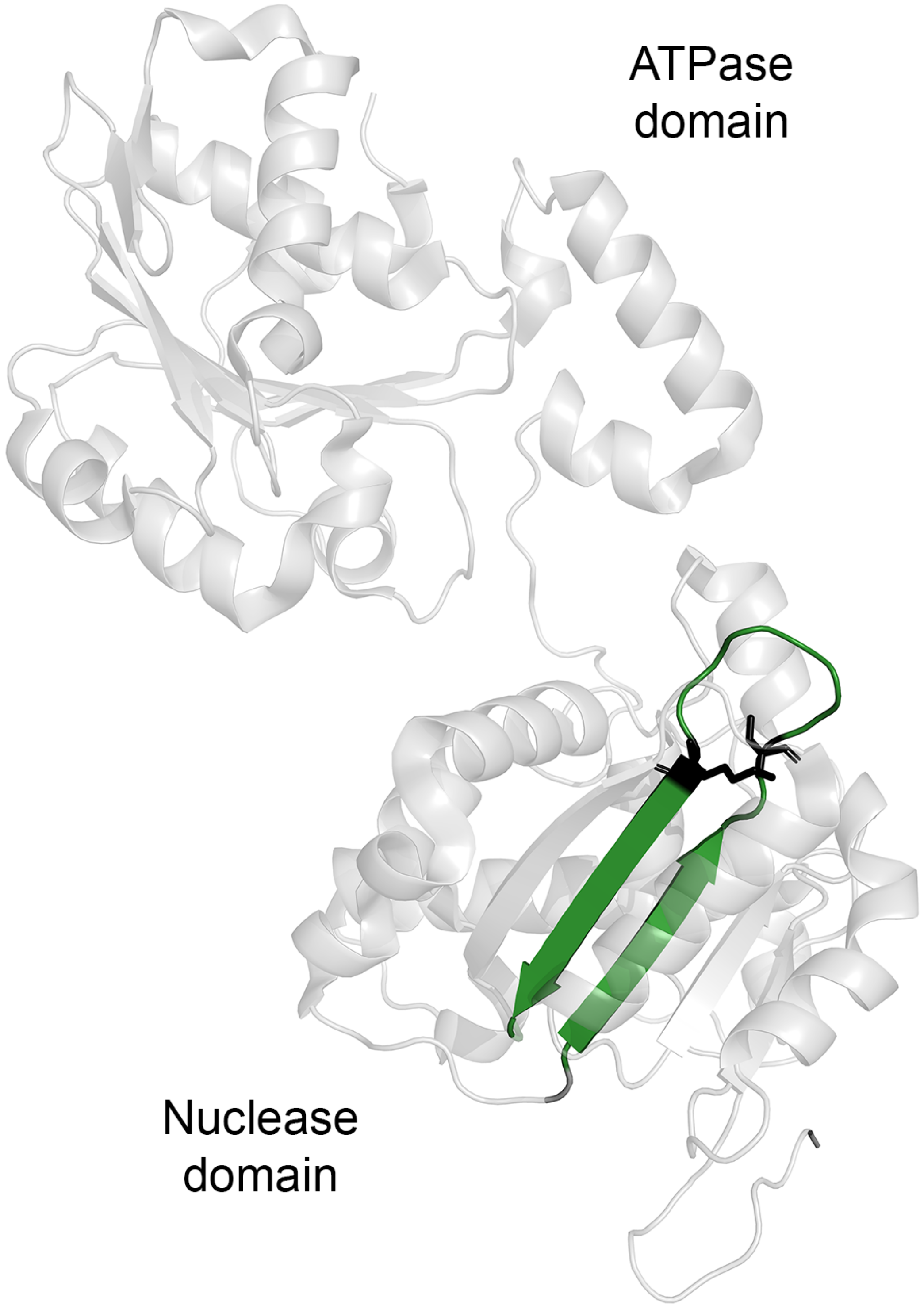

Supplementary Figure S2. Locations of the two conserved differentiating sites on the protein structure of the TerL from *Shigella* phage Sf6 (PDB ID 4IDH) (Zhao et al., 2013). The side chains of the two amino acids corresponding to the differentiating sites (S266 and K273) are shown in black. The two beta strands and a random coil region shown in **Supplementary Figure S1** are colored in green. The rest of the structure is shown at reduced opacity for presentation purposes.
